# Endothelial Cell Activation in an Embolic Ischemia-Reperfusion Injury Microfluidic Model

**DOI:** 10.3390/mi10120857

**Published:** 2019-12-06

**Authors:** Danielle Nemcovsky Amar, Mark Epshtein, Netanel Korin

**Affiliations:** Faculty of Biomedical Engineering, Technion-Israel Institute of Technology, Haifa 32000, Israel

**Keywords:** ischemia/reperfusion injury, thrombolysis, organ-on-a-chip, endothelial cell activation

## Abstract

Ischemia, lack of blood supply, is associated with a variety of life-threatening cardiovascular diseases, including acute ischemic stroke and myocardial infraction. While blood flow restoration is critical to prevent further damage, paradoxically, rapid reperfusion can increase tissue damage. A variety of animal models have been developed to investigate ischemia/reperfusion injury (IRI), however they do not fully recapitulate human physiology of IRI. Here, we present a microfluidic IRI model utilizing a vascular compartment comprising human endothelial cells, which can be obstructed via a human blood clot and then re-perfused via thrombolytic treatment. Using our model, a significant increase in the expression of the endothelial cell inflammatory surface receptors E-selectin and I-CAM1 was observed in response to embolic occlusion. Following the demonstration of clot lysis and reperfusion via treatment using a thrombolytic agent, a significant decrease in the number of adherent endothelial cells and an increase in I-CAM1 levels compared to embolic occluded models, where reperfusion was not established, was observed. Altogether, the presented model can be applied to allow better understanding of human embolic based IRI and potentially serve as a platform for the development of improved and new therapeutic approaches.

## 1. Introduction

Cardiovascular diseases are the leading cause of mortality in the Western world. According to the World Health Organization, (WHO), 17.9 million people die each year, representing 31% of all death worldwide [1]. Lack of proper blood supply, also known as ischemia, is associated with several medical conditions including myocardial infraction, thrombotic stroke, embolic vascular occlusion, angina pectoris, cardiac surgery and organ transplantation [2,3]. Although, restoration of blood supply is crucial to prevent further cellular damage, rapid reperfusion increases tissue damage, even in remote tissues that were not affected by the ischemia. This phenomenon where restoration of flow paradoxically increases tissue damage is termed ischemia reperfusion injury (IRI) [4,5].

In order to study IRI pathophysiology and to develop new therapeutic approaches a variety of IRI animal models have been employed [6,7,8]. These include both small animal models as well as large animal models, which better resemble human anatomy but are more costly. However, IRI animal models do not properly recapitulate critical components in the physiology of human ischemia reperfusion injury. As a result, despite the promising outcomes of some pharmacological treatments in IRI animal models, when these studies were implemented in human clinical trials the results were disappointing. For example, although statin treatments, which can reduce the inflammatory response in IRI, showed promising results in a rat IRI model [9], in human clinical studies, the results were less conclusive [10]. Thus, there is both a scientific need and an ethical motivation to develop alternative in vitro models of ischemia reperfusion injury, which would be relevant to human physiology.

Microfluidic cell culture devices, also known as organ-on-chips, offer an alternative system for recapitulation of certain features of human physiology and organ functionality in vitro. A variety of organ-on-chip platforms have been developed to study blood vessels, muscle, brain, liver and lung physiology [11]. Such microfluidic devices allow the culturing of human cells under a controlled microenvironment and well-defined dynamic conditions [12] and thus can be valuable for studying ischemia and IRI. A variety of microfluidic devices that precisely control oxygen levels and shear stress inside the device have been developed to study the role of these factors in health and disease conditions [13]. Abaci et al. studied the effect of these two factors under physiological conditions in a microfluidic vascular model and showed that the vasotoxic cancer drug, 5-Fluorouracil, induces vascular hyper-permeability and not apoptosis. Additionally, using their microfluidic model, they demonstrated the transition of endothelial cells from a quiescent to an activated state under hypoxic conditions. Khanal et al. studied IRI in cardiac tissue using a microfluidic device with porcine cardiomyocytes where the cells were subjected to nitrogen to induce hypoxia followed by normoxia; mitochondrial depolarization, cell adhesion and morphology were then assessed [14]. Despite the significant advances in the field, only limited work has focused on recapitulating IRI per se, where flow is rapidly restored following vascular occlusion. Additionally, thus far, to the best of our knowledge, no study has addressed embolic IRI, where the obstruction of flow is cause by a clot/embolus, and its lysis/removal may result in an IRI, as is the case in the treatment of ischemic stroke.

Here, we propose a microfluidic IRI model comprising a vascular compartment lined with human endothelial cells that can be obstructed with a human blood clot and then re-perfused via thrombolytic treatment. As vascular inflammation is an important symptom and contributor to IRI, we focused on endothelial cell activation, which is an important initial step in this process. Activated endothelial cells overexpress several receptors on their membrane, in order to attract immune cells, including E-selectin, which is responsible for leukocyte rolling, and I-CAM1, which is responsible for leukocytes firm adhesion to endothelial cells [15]. Using our model, we simulate treatment by a thrombolytic agent that, although restores flow to a clot-occluded vascular compartment, causes endothelial cell (EC) activation, as measured by the over-expression of the EC surface receptors E-selectin and I-CAM1. Thus, the presented model allows us to replicate, in a controlled manner, key features of IRI following restoration of flow upon removal of vascular embolic occlusion. Altogether, the suggested model may offer a better understanding of human IRI physiology in thromboembolic diseases and can also potentially serve as a novel platform to study new therapeutic approaches for treatment of IRI.

## 2. Materials and Methods 

### 2.1. Microfluidic Device

**Figure 1:**
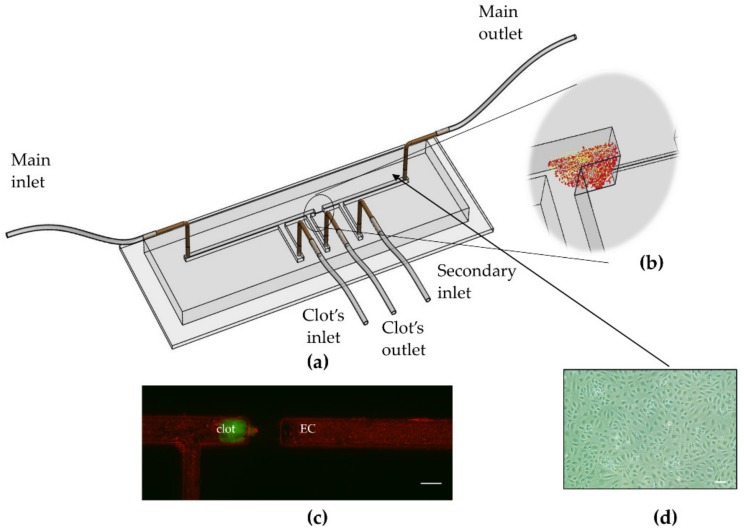
The microdevice recapitulating an embolic ischemia reperfusion injury. (**a**) Illustration of the microfluidic device: a narrowed section (200 µm × 200 µm) enables the occlusion by a clot prior to the vascular compartment, lined with endothelial cell. The different inlets and outlets allow independent perfusion of the cells and loading of the clot, as well as introduction of a thrombolytic solution to restore flow. (**b**) Schematic of the narrowed section in the microdevice where the blood clot obstructs the flow. (**c**) Live staining of endothelial cells (in red) and a blood clot occluding the channel (in green). Scale bar: 1 mm. (**d**) Phase microscopy image of the endothelial cells (ECs) inside the microfluidic device. Scale bar: 10 μm.

The microfluidic device was designed using SolidWorks. The design contains two straight channels, 1 mm in height and width and 3 cm in length, connected by a narrowing, 200 μm in height and width and 2 mm in length, between them designed to allow a blood clot to occlude and obstruct the normal flow. The microfluidic device has one main inlet for cell seeding and perfusion. A second inlet, the clot inlet, was added for clot injection and its corresponding outlet is just before the narrowing, in order to avoid flow through the EC compartment. Another inlet was added at the EC channel post the narrowing, for measurement purposes when the pre-stenosis channel is occluded with a blood clot. The microdevice mold was 3D printed (Object® Eden500, Formlabs, Massachusetts, USA) using Formlabs Clear Resin v4. Polydimethylsiloxane (PDMS, sylgard® 184, Silicone Elastomer) was poured over the mold and allowed to cure for 24 h, at room temperature. The PDMS was peeled from the mold and 1 mm holes were punched to create inlets and outlets. The PDMS and a glass slide were treated with plasma to create firm bonding and placed at 60 °C, overnight. A schematic view and image of the microdevice are shown in Figure 1a.

### 2.2. Cell Culture

Human umbilical vein endothelial cells (HUVECs, Lonza) were maintained in endothelial cell medium (ECM, ScienceCell, Carlsbad, CA, USA). Cells were cultured at 37 °C, in a humidified environment, with 5% CO_2_. Cells were passaged every 4–5 days, i.e., when they reached 85% confluence, using Trypsin EDTA solution B (Biological Industries, Cromwell, CT, USA). The microdevice was coated with human fibronectin (100 µg/mL, Sigma), overnight, at 4 °C. HUVECs at passages 4–6 were seeded in the microdevice at a density of 1.5 × 10^6^ cells/mL and incubated for two hours to allow the cells to adhere. The microdevice was connected to a programmable syringe pump for controlled perfusion overnight (flow rate 50 µL/h). Figure 1b shows a phase microscopy image of ECs grown under perfusion within the device.

### 2.3. Clot Fabrication

Calcium chloride (1 mol/L) was added to citrated human whole blood at a 5:1 ratio, to allow recalcification of the blood. Human fibrinogen plasminogen-depleted (5 mg/mL, Enzyme Research Laboratories) was added to stabilize the clot and increase its stiffness [16]. Human alpha-thrombin (1 u/mL, Enzyme Research Laboratories) was added to initiate clot formation. The solution was immediately injected into silicone tubes (Tygon, I.D. 0.79 mm) [17] and clots were incubated at 37 °C, overnight, to allow maturation. Clots were cut to 2 mm size in length and then incubated in saline for one hour prior to being inserted into model. Then a single clot was injected into the microfluidic device and allowed to occlude the entrance of the endothelialized vascular compartment (Figure 1c).

### 2.4. Thromblysis 

A blood clot was prepared as described above and infused into the main microchannel via the clot inlet it produced an occlusion of the entrance to the narrowed section. A thrombolytic solution was prepared by mixing human Glu-Plasminogen (200 µg/mL, Enzyme Research Laboratories) with serum free medium and tissue plasminogen activator (tPA, 126 µg/mL, Actilyse), a thrombolytic drug. The solution was infused into the device through the inlet and to the pre-stenosis outlet. The pre-stenosis outlet was then blocked and a constant pressure (60 mmHg) was enforced using the pressure-controlled MFCS^TM^-EZ system (Fluigent, Paris, France). The device was placed under an upright fluorescence microscope (Nikon SMZ25, Nikon Instruments Inc., Melveille, NY, USA) for time-lapse imaging. Flow rate was recorded during the experiment using a flow rate sensor (Fluigent, Paris, France).

### 2.5. Immunofluorescence

Following each experiment, the microdevice was washed three times with phosphate buffered saline (PBS) and then the cells were fixed with 4% paraformaldehyde (PFA), for 15 min, at room temperature. The cells were washed three times with PBS and incubated with blocking buffer (Bovine serum albumin 2%), for one hour. As E-selectin and I-CAM1 are receptors expressed on endothelial cell membrane and play an important role during inflammation. The E-selectin binding peptide (Esbp, CDITWDQLWDLMK–CONH2), that was synthesized by Shamay et al [18] and labeled with fluorescein isothiocyanate (FITC)-Lys. HUVECs were incubated with Esbp (50 µg/mL) for two hours at room temperature. To stain ICAM-1, cells were incubated at 4 °C, overnight with a mouse anti-ICAM-1 monoclonal antibody (5 μg/mL, ThermoFisher), which reacts with the human ICAM-1 receptor. The microdevice was then washed twice and incubated with a secondary antibody for labeling (Alexa Fluor 647 anti-mouse, ThermoFisher) for one hour. Lastly, cells were incubated for 5 min with 4’,6-Diamidino-2-Phenylindole, Dihydrochloride (DAPI), to stain the nucleus in all the channels. Cells were then washed three times with PBS.

### 2.6. Image Acquisition and Quantification

Images were taken with an inverted Eclipse Ti-E (Nikon Instruments Inc., Melville, NY, USA) microscope equipped with a 10× objective (N.A. 0.45, Nikon Instruments Inc., Melville, NY, USA) and an EMCCD camera (iXon Ultra, Andor-an Oxford Instruments company, Belfast, Ireland). ANDOR iQ3 Imaging software (Andor-an Oxford Instruments company, Belfast, Ireland) was used for stage movement control and image capture. Quantification of fluorescence intensity and number of cells was done with NIH ImageJ. 

### 2.7. Statistical Analysis

Data analysis was performed with a *t*-test: two sample assuming unequal variance. The amount of variation within the data set is presented by the standard deviation (SD), and the hypothesis test was conducted using *p*-value to determine the significance of the results. *p*-value < 0.05 is presented using (*), *p*-value < 0.01 (**), *p*-value < 0.001 (***) and *p*-value < 0.0001 (****). All experiments were repeated at least three times.

## 3. Results

### 3.1. Establishing Embolic Vascular Occlusion in the Device 

Microfluidic devices were coated with fibronectin and seeded with HUVECs. After seeding, cells were incubated for two hours to allow adherence. Cells were maintained under constant flow for 24 hours in order to achieve over 95% confluence and reduce any stress that can be induced by cell harvesting. The microfluidic device was designed to have a 200 µm narrowing between the two straight 1 mm channel compartments, thus allowing a pre-prepared human blood clot to obstruct and occlude the flow. Using separate inlet/out ports, we were able to incorporate the blood clot without harming the cell culture; >90% confluence was maintained (Figure 1).

### 3.2. Endothelial Activation upon Vascular Occlusion 

Both ischemia upon vascular occlusion and IRI, which follows upon restoration of flow are characterized by an inflammatory response in the affected tissue [19]. One of the key steps in the inflammatory process, which has also been a therapeutic target in IRI, is EC activation [20]. During EC activation, numerous receptors are overexpressed on the cell membrane [21], including E-selectin and I-CAM1. E-selectin is a glycoprotein expressed on endothelial cells after activation by numerous cytokines such as TNF-*α*. It is the main receptor on EC responsible for the initial adhesion of leukocytes during the inflammatory response [22]. I-CAM1 is a member of the immunoglobulin family, whose levels increase significantly following endothelial activation. It is the key mediator of the firm adhesion of leukocytes to endothelial cells [23]. To monitor E-selectin and ICAM-1 expression levels we used a fluorescence staining procedure, as outlined in the Methods section.

**Figure 2:**
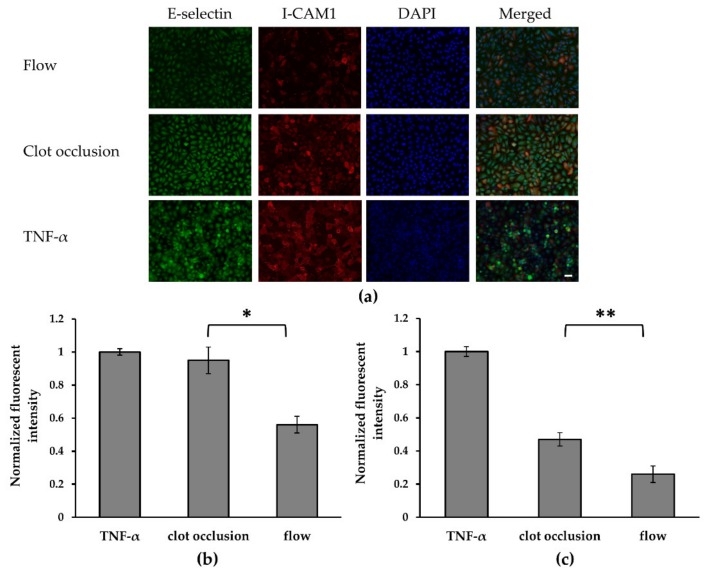
Endothelial cell (EC) activation following embolic occlusion in a microfluidic device. (**a**) Fluorescence confocal imaging of ECs stained for E-selectin, using Esbp (in green), I-CAM1, using a mouse-anti-ICAM1 antibody (in red) and nuclei (in blue), under various conditions (i) normal flow in the channel (FLOW), (ii) channel occluded with a blood clot (CLOT) or (iii) channel perfused with TNF-*α*, a proinflammatory stimulator (TNF-*α*). Scale bar: 10 μm. E-selectin and I-CAM1 intensity represents receptor expression, it elevates in correlation with cell activation. (**b**,**c**) Graph comparing between the normalized fluorescence intensity of (**b**) I-CAM1 and (**c**) E-selectin under the conditions mentioned in A. Both I-CAM1 and E-selectin levels in TNF- α channel and occluded channel increased compared to normally perfused channel with statistics significance. Significance determined by the unpaired Student’s *t*-test, *: *p*-value < 0.05, **: *p*-value < 0.01 (*n* = 3).

As a first step we tested whether embolic occlusion in our device results in EC activation. Staining of E-selectin and I-CAM1 was done following 2 h under the following conditions (a) channels perfused with serum free medium (unblocked), (b) channels obstructed by an emboli and (c) channels perfused with the inflammatory cytokine TNF-*α* at (0.1 µg/mL) in serum free media. TNF-α is known to induce a long-term inflammatory response by stimulating inflammatory mediators and proteases, thereby promoting endothelial cell activation [24]. As shown in Figure 2, indeed exposure to TNF-*α* caused a profound increase in the expression of both E-selectin (green) and I-CAM1 (red). Importantly, a significant increase in both receptors was measured in the embolic occlusion model compared to the results in the perfused devices. These results confirm that upon embolic occlusion in the designed device, the basal levels of both receptors increase, indicating EC activation. 

### 3.3. Restoration of Flow via Thrombolysis

**Figure 3:**
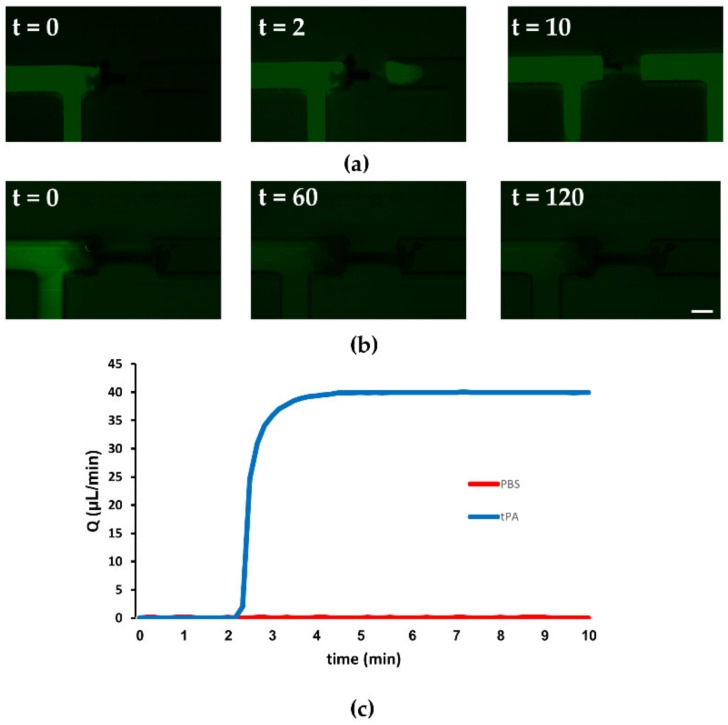
Thrombolytic reperfusion in the ischemia reperfusion injury (IRI) microfluidic device (**a**,**b**) Fluorescence time-lapse microscopy following thrombolysis and control treatment. (**a**) Treatment with a thrombolysis solution comprised of tissue plasminogen activator (tPA), plasminogen and FITC-dextran showing the reperfusion of a FITC-dextran solution upon treatment with tPA. (**b**) Treatment with phosphate buffered saline (PBS) with FITC-dextran (without tPA)—reopening did not occur even after two hours of experiment. Scale bar: 1 mm. (**c**) Flow rate measurements for the control (PBS) and thrombolysis (tPA) solutions, flow rate increased within several minutes of treatment in the channel treated with the thrombolytic solution, indicating reperfusion of the channel.

To regain perfusion, we prepared a thrombolytic solution composed of tPA and PLG supplemented with FITC-dextran for fluorescence labeling. tPA is a protease that converts PLG into plasmin (PLS). PLS is the final product in the thrombolysis cascade, that dissolves the fibrin mesh and ultimately the blood clot [25]. Currently the only Food and Drug Administration (FDA)-approved therapy for ischemic stroke is the alteplase, which is recombinant tPA [26]. The therapy involves the intravenous infusion of alteplase, to dissolve the clot. Although the treatment is critical it has a sever side effect, intracerebral hemorrhage (ICH) [27]. To simulate a thrombolytic treatment in our model, a blood clot was inserted, and occlusion of the vascular compartment was confirmed. Then to restore flow, a thrombolysis solution was introduced to the pre-occluded side; a physiological pressure gradient of 60 mmHg was maintained using a pressure-controlled system. Upon administration of the thrombolytic drug, within <10 min clot degradation occurred (Figure 3a), and flow was restored as measured using a flow sensor (Figure 3c). However, when the same saline solution with FITC-dextran excluding the tPA was injected, the clot remained stable (>2 h) under the same pressure gradient and no flow was re-established, see Figure 3b,c. Thus, these results demonstrated the ability to replicate thrombolysis-based reperfusion following an embolic occlusion.

### 3.4. Endothelial Cell Activation upon Reperfusion 

**Figure 4:**
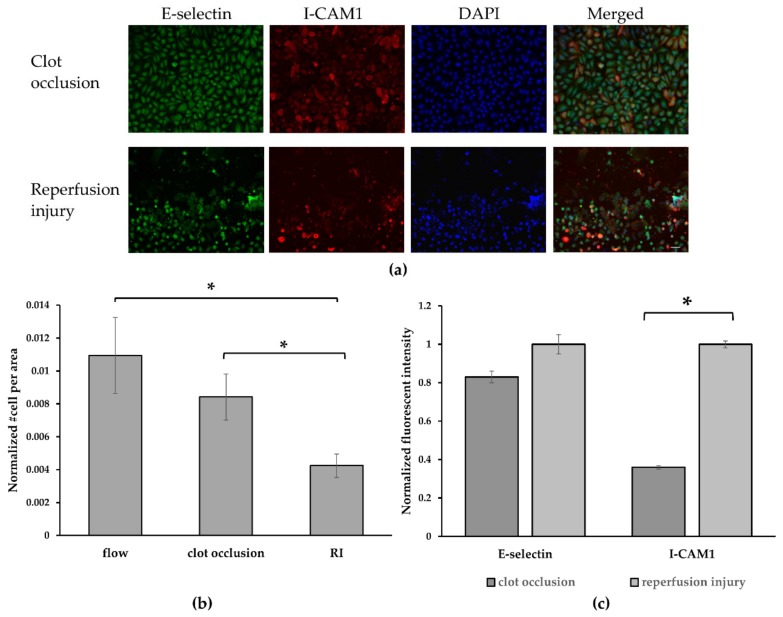
Reperfusion Injury and EC activation following reperfusion. (**a**) Confocal images of ECs stained for E-selectin (in green), I-CAM1 (in red) and nuclei (in blue). (i) Channel occluded with a blood clot. (ii) A re-perfused channel treated with tPA. Scale bar: 10 μm. (**b**) Normalized number of adherent cells per area. We can see significant decrease in number of adherent cells after reperfusion injury compared to normally perfused and occluded channels. (**c**) Normalized fluorescence intensity per cell of E-selectin and I-CAM1 in the occluded vs. the reperfused channels. Although not statistically significant is shown for E-selectin levels, for I-CAM the expression is significantly more pronounced. Statistical significance was determined by unpaired Student’s *t*-test *: *p*-value < 0.05 (*n* = 3).

Following our demonstration that reperfusion of an embolic occluded channel can be induced via a thrombolytic agent, we examined whether such a re-perfusion can result in EC activation and injury. As a first indication of EC injury following reperfusion, there was a significant decrease in the number of adherent ECs normalized per area, compared to the occluded channel and normally perfused channel see Figure 4a,b. This can be attributed to reperfusion injury, as the sudden flux of fluid could cause excessive trauma to the cells, which might result in their detachment. When EC activation was examined by quantitative evaluation of the E-selectin and I-CAM1 levels, normalized per cell, the results showed that although E-selectin levels were elevated to the same extent in both the re-perfused channel and the occluded channel, while I-CAM1 exhibited statistically significant higher expression in the re-perfused channel as compared to the occluded channel. These results implicate that indeed re-perfusion elevates EC activation as well as possible EC injury. 

## 4. Discussion

In this study, we examined an alternative in vitro microfluidic model to investigate, in a controlled manner, IRI following an embolic event. The model focused on simulating the injury to the endothelium, using a vascular compartment comprising human endothelial cells, which was subjected to an embolic occlusion and then to IRI as a result of restoration of flow via thrombolytic treatment. IRI is a highly complex condition, that involves proinflammatory and various pathophysiology processes. In this study, we focused on EC receptor overexpression as an indicator of EC activation, a known condition that occurs in IRI [28,29]. We confirmed in the microfluidic model that embolic vascular occlusion (>2 h) increased EC activation as compared to the perfused channel (Figure 2), as shown by significant elevation of both E-selectin and I-CAM1 levels. These results might be attributable to hypoxia or to other changes in the cellular micro-environment as a result of the occlusion and lack of an adequate medium supply. Additionally, experiment where reperfusion was established using a thrombolytic drug confirmed that reperfusion induced further cellular damage then caused by ischemia/occlusion. Under reperfusion, we noticed significant cell detachment from the microfluidic model and significantly increased I-CAM1 levels as compared to their levels in the ischemic channel. Although, E-selectin levels were elevated compared to normal flow, they increased to the same extent as they did during the occlusion conditions. This could be attributed to the fact the E-selectin is more sensitive to culture conditions and is also activated more rapidly, while I-CAM1 overexpression is a process that occurs over a long-time range and is more stable.

Although we focused on EC activation in the current study, other known IRI effects can be examined in our embolic IR model, including reactive oxygen species levels (ROS) [30], EC permeability [31], cell adhesion [32] and oxygen levels [33,34]. Additionally, while the current model focused on EC damage, use of a more complex co-culture model [31,35] could enable assessment of organ-specific tissue damage, such as the liver, brain and heart. Furthermore, we could combine multiple microfluidic devices with different cell types to study IRI on multiple organs simultaneously. Moreover, future studies could integrate blood components, known to play an active role in IRI, such as: leukocytes and platelets, which could be perfused into the system and their role might be studied.

While our results aligned with previous animal and in vitro studies [36,37,38], studies using rodent as animal model for IRI also showed an increase in the levels of ICAM-1 on EC membrane [37] and in leukocyte adhesion to EC after IRI [36]. Hence, we were able to recapitulate certain feature of IRI as seen in in vivo studies. Other in vitro model of IRI also exhibited higher levels of ICAM-1 after reperfusion [38]. However, this study induced ischemia in artificial manner. In this study we induced occlusion/ischemia by introducing an occlusive blood clot into the microfluidic device, in contrast to many animal and in vitro models. For example, in animal studies clamping of the artery is a very common way to mimic ischemia and IRI is induced upon removal of the clamp [36]. On the other hand, new in vitro studies in microfluidic devices have modulated the microenvironment inside the model (pH, hypoxia chambers, etc.) to achieve ischemic conditions [34]. However, to the best of our knowledge, no microfluidic study before has directly addressed the embolic IRI scenario. Additionally, with our approach, we might take into consideration the influence of the components relevant to the thrombolysis pathway as well as the kinetics of the thrombolysis process. Such a model could be beneficial for developing new therapeutic approaches as well as for studying ways to better perfuse occluded vessels while minimizing IRI in different clinical settings.
